# The Glymphatic System: A Novel Therapeutic Target for Stroke Treatment

**DOI:** 10.3389/fnagi.2021.689098

**Published:** 2021-07-08

**Authors:** Tao Lv, Bing Zhao, Qin Hu, Xiaohua Zhang

**Affiliations:** ^1^Department of Neurosurgery, Renji Hospital, School of Medicine, Shanghai Jiao Tong University, Shanghai, China; ^2^Central Laboratory, Renji Hospital, Shanghai Jiao Tong University School of Medicine, Shanghai, China

**Keywords:** glymphatic system, AQP4, astrocytes, meningeal lymphatic system, stroke

## Abstract

The glymphatic system (GS) is a novel defined brain-wide perivascular transit network between cerebrospinal fluid (CSF) and interstitial solutes that facilitates the clearance of brain metabolic wastes. The complicated network of the GS consists of the periarterial CSF influx pathway, astrocytes-mediated convective transport of fluid and solutes supported by AQP4 water channels, and perivenous efflux pathway. Recent researches indicate that the GS dysfunction is associated with various neurological disorders, including traumatic brain injury, hydrocephalus, epilepsy, migraine, and Alzheimer’s disease (AD). Meanwhile, the GS also plays a pivotal role in the pathophysiological process of stroke, including brain edema, blood–brain barrier (BBB) disruption, immune cell infiltration, neuroinflammation, and neuronal apoptosis. In this review, we illustrated the key anatomical structures of the GS, the relationship between the GS and the meningeal lymphatic system, the interaction between the GS and the BBB, and the crosstalk between astrocytes and other GS cellular components. In addition, we contributed to the current knowledge about the role of the GS in the pathology of stroke and the role of AQP4 in stroke. We further discussed the potential use of the GS in early risk assessment, diagnostics, prognostics, and therapeutics of stroke.

## Introduction

Clearing the metabolic wastes and maintaining the fluid homeostasis are important for brain function. In most organs, the lymphatic network is responsible for the wastes clearance and fluid drainage (Ikomi et al., 2012). However, a hallmark of the brain is the absence of typical lymphatic structures. Due to the presence of blood–brain barrier (BBB), the movement of solutes and ions in the brain is strictly restricted. Cerebrospinal fluid (CSF) has been considered to be important for the exchange of water-soluble metabolites; however, its mechanisms remain largely unknown. Iliff et al. (2012) reported the existence of the glymphatic system (GS) in the central nervous system (CNS), which is an alternative clearance system located in the perivascular space and aquaporin-4 (AQP4) dependent (Iliff et al., 2012). Emerging evidence from human studies and rodent models suggests that the GS is crucial for maintaining brain health, and dysfunction of GS is closely associated with various neurological disorders, including aging, neurodegeneration, and acute brain injury (de Leon et al., 2017; Ringstad et al., 2017). In parallel, the meningeal lymphatic vessels were discovered and demonstrated to participate in solutes transport and in immune surveillance (Aspelund et al., 2015; Louveau et al., 2015, 2016; Antila and Karaman, 2017).

Stroke, a major cause of death and disability, affects over 800,000 individuals annually (Coutts, 2017). It has been well-recognized that the GS plays a crucial role in the pathophysiology of stroke, including brain edema, blood–brain barrier (BBB) disruption, immune cell infiltration, neuroinflammation, and neuronal apoptosis (Ji et al., 2021). Targeting the GS, therefore, has provided potential for the early risk assessment, diagnosis, prognosis, and therapeutic of stroke. In this review, we summarize the latest research progress in the GS, including the anatomy and function, the interaction with the meningeal lymphatic systems and the BBB, and the communication between astrocytes and other GS cellular components. We emphasize the role of the GS in pathophysiology of different stroke subtypes, especially the role of AQP4 in the pathophysiology of stroke. In the end, we summarize the concerns and give some perspectives for future research.

## The Anatomy and Function of GS

**FIGURE 1:**
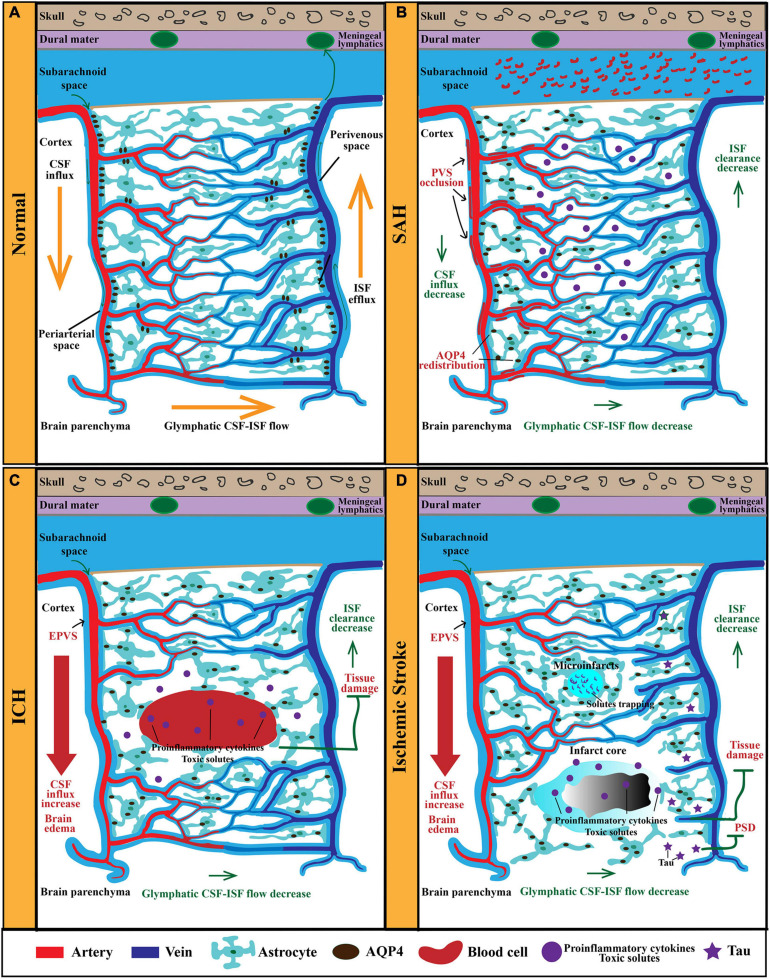
The anatomy and function of the GS in physiological and pathological conditions. **(A)** The GS mainly consists of periarterial CSF-inflow channel, perivenous ISF-outflow channel, and astrocytes-mediated convective transport of fluid and solutes. AQP4 polarized on astrocytic end-feet facilitates fluid and solutes exchange between the CSF and the brain interstitium. In physiological condition, CSF from the subarachnoid space is propelled into the brain parenchyma via the PVS of penetrating arteries. Then, CSF exchange with ISF in the extracellular space. Afterward, ISF and solutes move toward the perivenous space, ultimately drain out of the CNS via meningeal lymphatics. **(B)** After SAH, blood components invade the PVS rapidly, resulting in PVS occlusion and reduced CSF influx and ISF clearance. Furthermore, the perivascular polarity of AQP4 decreases after SAH, which resulted in accumulation of proinflammatory cytokines and neurotoxic solutes. In addition, AQP4 in the influx routes is upregulated markedly, while that in the efflux routes changes slightly. **(C)** In models of ICH, PVS is enlarged and responsible for brain edema. EPVS is also an independent risk factor for ICH recurrence. Moreover, glymphatic clearance rate is reduced, which contributes to the accumulation of proinflammatory cytokines and neurotoxic solutes. **(D)** In models of ischemic stroke, the ischemic spreading depolarizations along with subsequent vasoconstriction result in EPVS and doubled glymphatic inflow speeds. The increased influx of CSF in the GS contributes to poststroke edema. Additionally, the GS dysfunction after ischemic stroke impedes the clearance of neurotoxic solutes, proinflammation cytokines, and tau, which results in tissue damage and PSD. GS, glymphatic system; CSF, cerebrospinal fluid; ISF, interstitial fluid; PVS, perivascular space; CNS, central nervous system; SAH, subarachnoid hemorrhage; ICH, intracerebral hemorrhage; PSD, poststroke dementia.

The anatomy of the GS and its precise roles in fluid movement and drainage in the brain are complex. The GS is a glial-dependent fluid exchange and drainage system that comprises the entire perivascular space (PVS) network surrounding arteries, arterioles, capillaries, venules, and veins within the brain parenchyma. The PVS is constructed as a coaxial system where the inner cylinder is the cerebral vascular wall and the outer cylinder is the glial limitans that ensheathes the penetrating arterioles or perivascular astrocytic end-feet around the capillaries (Mathiisen et al., 2010). The GS mainly consists of periarterial CSF-inflow channel, perivenous ISF-outflow channel, and astrocytes-mediated convective transport of fluid and solutes supported by AQP4 water channels polarized on astrocytic end-feet (Benveniste et al., 2019). The patterns of fluid movement in the GS are similar to the classical Starling principle (Figure 1A), CSF from the subarachnoid space is propelled into the brain parenchyma via the PVS of penetrating arteries, also called Virchow–Robin space (Hannocks et al., 2018; Pizzo et al., 2018). CSF exchange with ISF then occurs through the glial basement membrane and astrocytic end-feet (Kress et al., 2014). From the interstitium, interstitial solutes and brain metabolic wastes flow into perivenous space and ultimately drain into lymph vessels existing in the meninges and perineural sheaths of cranial and spinal nerves to transport out of the CNS (Ma et al., 2017; Klarica et al., 2019). Currently, the glymphatic mechanism of solutes transport and wastes clearance is still unclear. The convective solutes transport and parenchymal diffusion transport are accepted by the majority of researchers (Smith et al., 2017; Smith and Verkman, 2018). What is particularly noteworthy is that the gap between astrocytic end-feet (gap width, 20–30 nm) traps larger molecular weight (MW) solutes and wastes during parenchymal CSF transport and drainage (Iliff et al., 2012). In addition, the GS is regulated by multiple factors, including arterial pulsations, respiratory pulsations, body position, and level of consciousness (Xie et al., 2013; Lee et al., 2015; Kiviniemi et al., 2016; Bedussi et al., 2018; Plog et al., 2019). However, there are limited investigations focusing on molecular mechanism that drives glymphatic fluid flow; more further studies are needed.

Furthermore, there is evidence for the crucial role of AQP4 in the GS. AQP4 is located in chromosome 18 (18q11.2–q12.1), and there is growing consensus that AQP4 polarized at the perivascular astrocytic end-feet facilitates glymphatic fluid transport and amyloid-β (Aβ) export in rodents. Iliff et al. (2012) indicated that glymphatic clearance was significantly reduced in AQP4 knockout mice compared with that in normal littermates. Several independent groups have replicated the initial finding of AQP4-dependent glymphatic clearance and the crucial role of AQP4 in facilitating Aβ clearance (Xia et al., 2017; Mestre and Hablitz, 2018). Recent study confirmed that altered localization of AQP4 in aged rodent brain resulted in significantly increased parenchymal retention of adeno-associated viruses (AAV) vectors, which supported the importance of AQP4 in facilitating efficient glymphatic transport and clearance (Murlidharan et al., 2016). However, Smith et al. (2017) observed glymphatic clearance in AQP4 knockout rodent brain parenchyma and questioned the importance of AQP4 in the GS. Currently, except AQP4, no other astrocytic ion channels have been reported to be involved in the functional regulation of GS.

Another controversial topic is the drainage routes out of the CNS. Previous studies have found that perineural sheaths of the olfactory nerve passing through the cribriform plate and ultimately flowing into the cervical lymphatic vessels are primary solutes and wastes egress routes in both rodents and humans (Ma et al., 2017). Previous studies found that surgical blockade of the perineural cribriform pathway contributed to a rapid and sustained increase in intracranial pressure (Mollanji et al., 2002). Over the past few years, emerging studies have shown that the meningeal lymphatic systems are involved in the drainage of glymphatic fluid, and the anatomy and function of this drainage pathway are being defined (Aspelund et al., 2015; Louveau et al., 2015; Antila and Karaman, 2017; Da Mesquita et al., 2018; Ahn et al., 2019; Esposito et al., 2019; Song et al., 2020). Therefore, we will discuss its association with the GS in the following sections.

## Glymphatics–Meningeal Lymphatics Connection

The meninges are composed of three layers: pia, arachnoid, and dura. Initially, similar to brain parenchyma, the meninges were long suggested to be devoid of lymphatic vessels. Aspelund et al. (2015) first described the structural and functional features of meningeal lymphatics and demonstrated that meningeal lymphatic vessels were mainly in the dura mater and well developed around the venous sinuses. Over the past decade, emerging evidence suggests that the meningeal lymphatics play a crucial role in macromolecular clearance, immune surveillance, and export of CSF/ISF from the CNS (Ahn et al., 2019; Alvesx de Lima et al., 2020; Song et al., 2020; Wojciechowski et al., 2020; Ding et al., 2021; Graham and Mellinghoff, 2021). Malfunction of meningeal lymphatics may cause the accumulation of toxic Aβ, cellular debris, inflammatory mediators, and immune cells, eventually resulting in neurological dysfunction and impacting neurological disease progression such as AD, traumatic brain injury, and subarachnoid hemorrhage (Da Mesquita et al., 2018; Bolte and Dutta, 2020; Chen and Wang, 2020; Mentis et al., 2021).

To elucidate the connection between the meningeal lymphatics and the GS, Louveau et al. (2015) administered fluorescent tracers into brain parenchyma and ventricles and observed tracers primarily along the meningeal lymphatic vessels that ultimately flowed into deep cervical lymph nodes (dcLNs). Furthermore, surgical ligation of the cervical lymphatic vessels enhanced tracers accumulation into the meningeal lymphatic network and prevented tracers from retention in dcLNs. These findings are supported by multiple studies using MRI and diffusion of radiolabeled tracers (Eide and Vatnehol, 2018; Alvesx de Lima et al., 2020; Zhou et al., 2020; Wu et al., 2021). These observations further indicate that meningeal lymphatics are the major efflux route for macromolecules, immune cells, and CSF/ISF. However, the exact proportions of different downstream routes of the GS, including meningeal lymphatics, perineural cribriform, and rostral migratory stream, are unknown (Goldmann et al., 2006; Kaminski et al., 2012; Ratnam et al., 2019). Hence, further investigation is required to elucidate the contribution and function of the different paths under both physiological and pathological statuses and to eventually understand how the GS links to downstream routes to maintain cerebral homeostasis.

In brain parenchyma clearance systems, besides the GS, other clearance mechanisms also have been observed, such as endocytosis and phagocytosis by pericytes, and transvascular clearance modulated by low-density lipoprotein receptor-related protein 1 (LRP1) (Kanekiyo and Bu, 2014). Indeed, studies have reported that BBB-associated pericytes could acquire a microglial phenotype following ischemic stroke (Özen et al., 2014). Ischemic brain injury increased the expression of microglial markers: IBA1, CD11b, and CD68 enhanced phagocytosis ability of pericytes to clear compromised cells (Özen et al., 2014). Pericytes also facilitate the clearance of soluble Aβ mediated by LRP1 (Ma et al., 2018). Moreover, it has been confirmed that brain endothelium enhanced the clearance of soluble brain Aβ in an LRP1-dependent manner (Storck et al., 2016). It is essential for these clearance systems to work in symbiosis to ensure the proper transport of solutes and brain homeostasis. In the future, further investigations should address the precise role of each clearance system in physiological and pathophysiological conditions and focus on the association of clearance systems with the meningeal lymphatics.

## Interaction Between the GS and the BBB

The GS is anatomically and functionally interconnected with the BBB, and together, they regulate the transport and exchange of fluid and solutes throughout the brain, thus maintaining brain homeostasis (Braun and Iliff, 2020).

**FIGURE 2:**
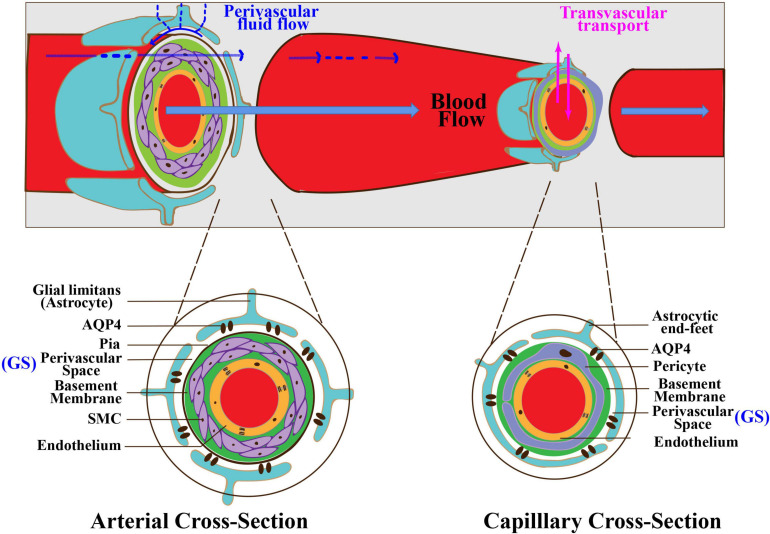
Interaction between the GS and the BBB. The GS is anatomically and functionally interconnected with the BBB. There is some overlap in anatomical structures between the BBB and the GS. At the arterial level (left inset), endothelial cells form the inner layer of the vascular wall. The basement membrane separates endothelium from SMCs. The basement membrane and SMCs are enveloped by the pia. The PVS is between the pia and the glia limitans formed by astrocytic end-feet. At the capillary level (right inset), pericytes and endothelial cells share a basement membrane. The PVS is between the basement membrane and astrocytic end-feet. The BBB regulates the exchange of molecules between the blood and the brain tissue via multiple transport systems. The GS regulates the exchange of fluid and solutes between the CSF and the ISF. GS, glymphatic system; BBB, blood–brain barrier; PVS, perivascular space; SMCs, smooth muscle cells.

The BBB is a multicellular vascular structure that insulates the neural tissue from the peripheral blood circulation. The BBB is composed of capillary endothelial cells, tight junctions between the adjacent endothelial cells, basement membrane, mural cells including pericytes and smooth muscle cells (SMCs), and astrocytes (Figure 2; Abbott et al., 2010). As mentioned above, the GS is a coaxial system that comprises the entire PVS network surrounding cerebral vessels. The inner cylinder of the GS is the cerebral vascular wall, while the outer cylinder is the glial limitans that ensheathes the penetrating arterioles or perivascular astrocytic end-feet around capillaries (Figure 2). Hence, there is some overlap in anatomical structures between the BBB and the GS. It is worth noting that BBB is a lateral physiological structure and places more emphasis on the selective permeability of substances, while the GS is a longitudinal physiological structure and places more emphasis on influx and efflux of CSF (Troili et al., 2020). Additionally, astrocytes play an essential role in both the BBB and the GS. Astrocytes take part in BBB maturation and maintenance by providing additional support such as perivascular astrocytic end-feet around capillaries and glial limitans around penetrating arterioles (Sweeney et al., 2019). In addition, astrocytes strengthen the tight junctions by regulating gene expression in endothelial cells via astrocyte–endothelial SHH pathway (Abbott et al., 2006). Astrocytes also enhance the basement membrane by producing laminin and stabilize pericytes via apolipoprotein E (ApoE) and low-density lipoprotein receptor-related protein 1 (LRP1) pathway (Bell et al., 2012). In the GS, astrocytes comprise the outer cylinder by astrocytic end-feet and glial limitans. Furthermore, AQP4-polarized astrocytes are necessary for the CSF/ISF exchange.

The BBB regulates molecules in blood transported into and out of the brain tissue and prevents blood cells, neurotoxic plasma components, and pathogens from entering the brain tissue. Currently, several kinds of transport system presented in the BBB have been identified, such as carbohydrate transporters, amino acid transporters, monocarboxylate transporters, hormone transporters, fatty acid transporters, nucleotide transporters, and organic anion and cation transporters (Sweeney et al., 2019). These transport systems are regulated mainly based on brain endothelial cells and vascular pericytes. The GS also regulates the exchange of solutes, but this mainly occurs between CSF and brain tissue. The transport and exchange of molecules in the GS are mainly dependent on the gap between astrocytic end-feet and AQP4 polarized on astrocytic end-feet (Iliff et al., 2012). Meanwhile, both the BBB and the GS are critical for the clearance of metabolic wastes in the brain (Ueno et al., 2019). Metabolic wastes or neurotoxic substances in the brain parenchyma could be eliminated by flowing into the blood through efflux transporters at the BBB to the blood or ISF bulk flow clearance of the GS through PVS to the cervical lymph nodes (Ueno et al., 2019). The BBB and the GS may cooperatively play a significant role in the maintenance of cerebral homeostasis (Braun and Iliff, 2020). BBB breakdown will cause alteration of cell polarity and change in transport mechanisms, ultimately aggravating the GS dysfunction. Disruption in the GS will lead to the obstruction of drainage space, metabolite imbalance, and accumulation of toxic substances, which, in turn, deteriorates the BBB structurally and functionally. One recent study enrolled 109 participants with cerebral small vessel disease (cSVD) and explored the association of the GS dysfunction with BBB integrity (Li et al., 2019). The results demonstrated that high-grade enlarged PVS (EPVS) and the GS dysfunction were associated with a higher BBB leakage rate, supporting the hypothesis that the GS dysfunction is part of the pathological processes of compromised BBB integrity. Collectively, further studies are needed to verify the causal relationship between the increased BBB permeability and the GS dysfunction.

## Communication Between the GS Cellular Components

The major cellular components of the GS are astrocytes but also include other cells, such as neurons, microglia, and pericytes. Astrocytes constitute the physical barrier of the GS with their end-feet processes in the PVS (Filosa et al., 2016) and regulate the exchange and clearance of solutes between CSF and ISF through AQP4 localized on its end-feet. Up to 50% of the brain AQP4 is expressed on the astrocytic end-feet, which supported the key role of astrocytes in the GS (Hubbard et al., 2018; Plog and Nedergaard, 2018; Yin et al., 2018). Through communicating with neurons, microglia, and pericytes, astrocytes regulate synaptic transmission, modulate microglial phenotypes, and control fluid and ion homeostasis in physiological and pathological conditions of the CNS.

### Astrocytes and Neurons Interactions

Astrocytes communicate closely with neurons. Previously, astrocytes were considered simply as a supportive function for neurons. Astrocytes protect neurons by releasing neurotrophic factors and antioxidants and by eliminating neuronal wastes (Wang et al., 2006). Astrocytes play a crucial role in synaptic transmission. It is estimated that a single astrocyte interacts with over 100,000 synapses, suggesting the close association between astrocytes and neurons (Bunney et al., 2017). Astrocytes also modulate neurotransmitter homeostasis through wrapping around presynapses and postsynapses to form a tripartite synapse (Allen and Barres, 2009; Sultan et al., 2015). Astrocytes also promote the neurogenesis via secreting D-serine (Sultan et al., 2013; MacKay et al., 2019), lactate (Zhou et al., 2019), interleukin-6 (IL-6) (Erta et al., 2015), interleukin-1 beta (IL-1β) (Jones et al., 2018), and growth factors, such as brain-derived neurotrophic factor (BDNF), fibroblast growth factor 2 (FGF-2), glial cell line-derived neurotrophic factor (GDNF), and vascular endothelial growth factor (VEGF) (Araki and Ikegaya, 2020). A recent research from rodent middle cerebral artery occlusion (MCAO) models demonstrates that, although astrocytes are not as mobile as microglia, they are able to polarize their distal processes without migration of cell body and phagocytose apoptotic bodies derived from dendrites of dying neurons in the infarct core (Damisah and Hill, 2020). What is more, a recent study demonstrated that decreased expression of astrocytic AQP4 accelerated deposition of α-synuclein and aggravated the loss of dopamine neurons via impairment of the GS (Cui et al., 2021). The decreased expression of astrocytic AQP4 is also associated with reduced dendritic spine density of cortical neurons (Venkat et al., 2017). Moreover, it has been proven that the depolarization of astrocytic AQP4 contributed to motor neuron degeneration (Dai et al., 2017).

In turn, astrocytes respond to the stimulation from neurons. Neurons, as a pacemaker of the neurovascular unit, detect very slight variations in nutrients and oxygen and then translate these changes into electrical signals and chemical messages to adjacent astrocytes (Figley and Stroman, 2011). It has been reported that astrocytic end-feet covered more than the 99% surface of cerebral blood vessels (Filosa et al., 2016). Neurons communicate with vessels via astrocytes, thus impacting the vascular tone and adjusting regional cerebral blood flow to provide proper supply of oxygen and nutrients (Muoio et al., 2014). Recent researches demonstrated that a high prevalence of pathological mitochondria in neurons increased the degree of astrogliosis and reduced the perivascular expression of AQP4 (Hasan-Olive et al., 2019). In general, further investigations that report the effect of neuron damage on the polarization of astrocytic AQP4 and the role of astrocytes in the GS are necessary.

### Astrocytes and Microglia Interactions

Microglia comprise 5–10% of the brain cells and act as resident immune cells in the CNS (Frost and Schafer, 2016). There are two phenotypes being identified for microglia: M1 phenotype characterized by producing inflammatory mediators, including tumor necrosis factor (TNF), IL-1β, and reactive oxygen species (ROS), and M2 phenotype characterized by secreting anti-inflammatory mediators, including interleukin IL-10, transforming growth factor beta (TGFβ), and glucocorticoids (Orihuela et al., 2016). It has been reported that the interaction between activated microglia and reactive astrocytes plays an important role in neuroinflammation following stroke (Magaki et al., 2018). Microglia are activated via responding to pathogens and secreting cytokines, such as interleukin-1 alpha (IL-1α), TNF-α, and the complement component subunit 1q (C1q), thus triggering astrocyte reactions (Liddelow et al., 2017; Yun et al., 2018). In the intracerebral hemorrhage (ICH) mice model, microglial activation in the perihematomal region is found within 1 h after ICH was induced. Molecular signals produced by M1 phenotype, such as IL-1β, TNF, and IL-6, and inducible nitric oxide synthase (iNOS) are amplified both in human and rodent perihematomal brain tissues (Clausen et al., 2008; Wu et al., 2010; Liesz et al., 2011; Takaoka et al., 2016). Similarly, in the MCAO mice model, microglia are activated and invade the infarct core, simultaneously secreting cytokines IL-1β, TNF, and IL-1 receptor antagonist (IL-1Ra) (Clausen et al., 2008, 2016; Michelucci et al., 2009). In addition, microglia enhance astrocytes responses via Toll-like receptor 4 (TLR4) activation under insults, injury, or inflammation in stroke (Holm et al., 2012). Notably, although reactive astrocytes triggered by microglia activation are harmful to their function in the GS, an enhanced uptake and phagocytosis of astrocyte solutes and microglia may offset the impairment of glymphatic clearance (Feng et al., 2020).

### Astrocytes and Pericytes Interactions

Pericytes are the main mural cells that maintain the BBB integrity at the capillary level (Jeske et al., 2020; Zhang et al., 2020e). In the brain, astrocytes–pericytes interactions play a key role in regulating BBB permeability and cerebral blood flow and helping to facilitate the clearance of toxic substances. Astrocytes are the main cells expressing ApoE in the brain (Zhao et al., 2017). Previous studies demonstrated that ApoE4 astrocytes reduced Aβ clearance and increased cholesterol accumulation compared with ApoE3 astrocytes (Lin et al., 2018; Prasad and Rao, 2018). In addition, ApoE4 astrocytes aggravate pericytes degeneration and BBB breakdown via activating the cyclophylin A–nuclear factor kappa B (NF-κB)–matrix metalloproteinase 9 pathway in pericytes (Halliday et al., 2016; Sweeney et al., 2016). Furthermore, astrocytes promote cell contraction by increasing intracellular Ca^2+^ or K^+^ transients in pericytes (Mishra et al., 2016; Sweeney et al., 2016). Astrocytes have been reported to secrete prostaglandin E2 (PGE2) and activate PGE2 receptor 4 in pericytes to induce pericytes relaxation (MacVicar and Newman, 2015). Astrocytes regulate cell differentiation and maintain BBB integrity via secreting laminin α-2 chain (Lamα2) and interact with integrin α-2 receptor in pericytes (Yao et al., 2014). Conversely, pericytes regulate the polarization of astrocytic AQP4 (Gautam et al., 2020). In pathological conditions, pericyte deficiency reduces the expression of α-syntrophin, the component of dystrophin complex that regulates AQP4 anchoring, resulting in a redistribution of AQP4 on astrocytic end-feet where AQP4 moves away and is expressed onsite without vessel contact (Anderova et al., 2014; Gundersen et al., 2014).

## The GS Dysfunction in Stroke

The GS dysfunction has been demonstrated to be involved in the pathophysiology of brain edema, BBB disruption, neuroinflammation, and neuronal cell death after stroke. In this section, we summarize the new discoveries about function of the GS in pathophysiological process of hemorrhagic and ischemic stroke (Table 1).

**TABLE 1:** The researches focused on the role of the GS in stroke.

**Article**	**Model**	**Species**	**Key details of study**
SAH			
Gaberel et al., 2014	Autologous arterial blood injection in the prechiasmatic cistern	Mice	• PVS was occluded by fibrin clots and the GS was severely impaired.
Luo et al., 2016	Autologous arterial blood injection in the cisterna magna	Mice	• Blood components invaded brain parenchyma along the PVS. • Blood components in the PVS induced neuroinflammation of perivascular parenchyma. • The CSF circulation in brain parenchyma was severely impaired.
Golanov et al., 2018	Endovascular perforation	Rats	• CSF flow along the GS was interrupted for up to 30 days after SAH
Goulay et al., 2017	Autologous arterial blood injection in the optic cistern	Non-human primate	• The fibrin and fibrinogen were deposited in the PVS. • The CSF circulation in brain parenchyma was severely impaired.
Pu et al., 2019	Autologous arterial blood injection in the cisterna magna	Mice	• The polarization of astrocytic AQP4 and the GS were impaired, which resulted in accumulation of Tau proteins and CD3+, CD4+, and CD8+ cells in brain parenchyma.
Liu et al., 2020b	Endovascular perforation	Rats	• AQP4 knockout aggravated brain edema, BBB disruption and neuronal apoptosis. • SAH induction in AQP4 deficit rat significantly impaired ISF transportation.
Liu et al., 2020a	Endovascular perforation	Rats	• The inflow of CSF into the brain and the clearance of ISF from the brain were both markedly decreased. • The expression level of AQP4 around the artery was higher than that around the vein after SAH. • AQP4 knockout aggravated the GS damage after SAH.
Garland et al., 2021	/	Human	• The GS dysfunction following SAH resulted in accumulation of neurofilament light.
ICH			
Duperron et al., 2019	/	Human	• The increased EPVS burden was associated with incidence of ICH.
Raposo et al., 2019	/	Human	• The increased EPVS burden in the centrum semiovale was linked to vascular amyloid burden after acute ICH.
Raposo and Viswanathan, 2020	/	Human	• EPVS was associated with recurrence of ICH.
Best et al., 2020	/	Human	• EPVS was an independent risk factor for symptomatic ICH in patients receiving OAC.
Ischemic stroke			
Gaberel et al., 2014	MCAO	Mice	• Glymphatic perfusion poststroke was markedly impaired.
Wang et al., 2017	Intraarterial injection of cholesterol crystals	Mice	• The GS around microinfarcts was focally disrupted. ∙The impairment of glymphatic clearance led to neuroinflammation and neuronal apoptosis.
Zbesko et al., 2018	MCAO	Mice	• Neurotoxic solutes and proinflammation cytokines were trapped in infarct core. • The extracellular fluid in infarct core was long-lasting toxic due to the impairment of glymphaticclearance.
Back et al., 2020	MCAO + BCCAO	Rats	• Astrocytic AQP4 distribution changed from perivascular to brain parenchyma. • The GS dysfunction resulted in impairment of tau clearance and PSD.
He et al., 2020	Laser-evoked arteriole occlusion	Mice	• Slit2 facilitated glymphatic clearance and improved cognition in microinfarct model.
Lin et al., 2020	MCAO	Rats	• The influx of CSF was slow even 7 days after ischemic stroke.
Mestre and Du, 2020	MCAO	Mice	• The influx of CSF increased rapidly within minutes after ischemic stroke.
Zhang et al., 2020d	/	Human	• The increased EPVS burden was associated with a higher risk of ischemic stroke.
Bu et al., 2021	/	Human	• The increased EPVS burden was associated with a higher risk of ischemic stroke.

*BCCAO, bilateral common carotid artery occlusion; EPVS, enlarged PVS; GS, glymphatic system; ICH, intracerebral hemorrhage; MCAO, middle cerebral artery occlusion; OAC, oral anticoagulant; PSD, poststroke dementia; PVS, perivascular space; SAH, subarachnoid hemorrhage; Slit2, slit homolog 2.*

### Subarachnoid Hemorrhage

Subarachnoid hemorrhage (SAH) is a devastating form of stroke often with permanent brain impairment. Currently, there is no effective intervention for preventing secondary neuropathological damage and improving the prognosis of SAH patients. Impaired CSF circulation along periarterial influx routes has been indicated in rodent models and gyrencephalic non-human primate model of SAH and has shed new light on translational therapeutic strategies (Gaberel et al., 2014; Goulay et al., 2017; Golanov et al., 2018; Liu et al., 2020a,b; Figure 1B). Following SAH, blood components particularly fibrin and fibrinogen deposit in PVS, which led to occlusion and dysfunction of the GS, ultimately worsening cerebral ischemia and edema (Goulay et al., 2017). Further studies confirmed that subarachnoid blood invaded the PVS within 5 min after SAH induction, gradually penetrating into the brain parenchyma in the following hours (Luo et al., 2016). They further demonstrated that the GS dysfunction following SAH resulted in vasculitis, widespread microinfarction, and neuroinflammation (Luo et al., 2016). In a more recent study, Pu et al. (2019) injected fluorescent tracers into the cisterna magna of SAH mice and found that the tracers that flowed into the brain parenchyma and drained to the dcLNs were significantly reduced after SAH induction. A further study found that the CSF influx into the brain and the ISF egress from the brain both significantly decreased after SAH (Liu et al., 2020a). In non-human primates, Goulay et al. (2017) injected gadolinium chelate into the cisterna magna and evaluated the parenchymal CSF circulation in healthy or SAH induction status using living MRI. They found a gradual active distribution of CSF from cerebral ventricles to the superficial part of the brain and then to deeper structures of the brain in normal physiological status, whereas SAH induction significantly impaired parenchymal CSF circulation (Goulay et al., 2017). Collectively, these observations support the evidence that GS dysfunction is an important pathophysiological feature and associated with secondary brain injury and neurological deficits following SAH. Interestingly, it has been reported that clearance of the PVS with tissue-type plasminogen activator could alleviate histological injury and improve behavioral deficits of SAH (Luo et al., 2016; Bosche and Mergenthaler, 2020). Therefore, targeting the GS potentially serves as a novel strategy for the treatment of SAH. However, further studies are necessary to investigate the underlying molecular mechanisms governing the link between SAH and the GS.

### Intracerebral Hemorrhage

Intracerebral hemorrhage (ICH) is another severe hemorrhagic stroke subtype with high risk of death and disability (Charidimou et al., 2017). The research related to the association of the GS with ICH is limited. Several studies reported the role of EPVS in the pathology of ICH. As mentioned above, PVS is a key anatomical structure of the GS. Normally, the diameter cutoff of PVS is set as 3 mm (Zhu et al., 2011; Wardlaw et al., 2013, 2020; Dubost et al., 2019). Previous studies demonstrated that EPVS was linked to impaired glymphatic clearance (Mestre et al., 2017; Boespflug et al., 2018; Opel et al., 2019). The GS dysfunction contributes to the accumulation of metabolic wastes and neurotoxic substances in PVS, ultimately resulting in EPVS (Figure 1C). Therefore, EPVS is a marker of the GS dysfunction. EPVS has been indicated to be associated with perforator arteriopathy and emerged as a marker of small vessel disease (SVD) (Charidimou et al., 2017; Brown et al., 2018; Jokinen et al., 2020). Recent studies provide new evidence that EPVS is linked to an increased risk of ICH. One study followed 1,678 participants for 10 years and found that increasing global EPVS burden was associated with a higher risk of incident ICH (Duperron et al., 2019). In another study, researchers included 1,386 patients with atrial fibrillation receiving oral anticoagulants (OAC) after a recent transient ischemic attack (TIA) or ischemic stroke and followed subjects for a mean of 2.3 years while assessing EPVS with MRI and found that EPVS was an independent risk factor for symptomatic ICH in patients receiving OAC (Best et al., 2020). However, the underlying mechanisms leading to the transformation from EPVS to symptomatic ICH remain unclear. Recent research has further demonstrated that EPVS was associated with ICH recurrence (Raposo and Viswanathan, 2020). During the recovery process of ICH, one study examined the connection between EPVS and Aβ deposition through MRI and 18F-florbetapir PET, respectively, and demonstrated that EPVS that appears in the centrum semiovale was the marker of vascular Aβ burden and enhanced the risk of poor prognosis (Raposo et al., 2019). Therefore, there is no doubt that EPVS is of great clinical significance in the pathology of ICH, and further investigations need to illustrate the molecular mechanism of EPVS aggravating the pathological process of ICH.

### Ischemic Stroke

Findings from the current studies indicate that the GS is involved in the pathology of ischemic stroke (Figure 1D). First, the GS dysfunction is a predictor of incidence of ischemic stroke. According to accumulating brain MRI and pathological studies, EPVS has been identified as the risk of stroke (Zhang et al., 2020d; Bu et al., 2021).

Second, CSF circulation is impaired after ischemic stroke, although there is still lack of consensus on the dynamic change in CSF flow in the GS. In murine models of acute ischemic stroke (AIS), CSF inflow in the ipsilateral cortex has been verified to be impaired at 3 h after AIS induction via MRI and histological examination (Gaberel et al., 2014). Consistently, researchers found that the influx of CSF was slow even 7 days after ischemic stroke (Lin et al., 2020). However, a recent study found a conflicting conclusion; researchers discovered that the influx of CSF in the GS increased rapidly within minutes of an ischemic insult (Mestre and Du, 2020). The ischemic spreading depolarizations along with subsequent vasoconstriction might be responsible for EPVS and doubled glymphatic inflow speeds (Mestre and Du, 2020). Meanwhile, the increased CSF flow in the GS was considered to be the primary cause of acute ischemic tissue swelling (Mestre and Du, 2020). These findings revised our understanding of poststroke edema.

Notably, the reduced glymphatic clearance after ischemic stroke is defined. Researchers injected fluorescent tracers into the infarction area of a rodent MCAO stereotactically to evaluate the clearance of solutes, and the results showed that tracers were trapped in the infarct core (Zbesko et al., 2018). Further results from Yang et al. (2015) are consistent with this finding (Lin et al., 2020). They used contrast-enhanced MRI by injecting Gd-DTPA into the cisterna magna to assess the GS function in the acute and subacute phases of ischemic stroke induced by MCAO. They evaluated the time course of the signal-to-noise ratio (SNR) in the substantia nigra (SNe) and ventral thalamic nucleus (VTN) and found that the SNR time-to-peak on the ipsilateral side was longer in SN both in the acute phase and in the subacute phase than in the contralateral phase (Lin et al., 2020). In addition, the GS dysfunction contributes to the accumulation of toxic solutes and proinflammatory cytokines within the core infarction area. It is well known that damaged brain tissue after AIS goes through the stage of liquefactive necrosis, which produces poisonous extracellular fluid. One study demonstrated that extracellular fluid present in areas of liquefactive necrosis following ischemic stroke was injurious to primary cultured cortical and hippocampal neurons even after 7 weeks following stroke (Zbesko et al., 2018). Interestingly, a recent study found that overexpression of slit2 alleviated neuronal excitotoxicity and improved cognition via accelerating glymphatic clearance after ischemic stroke (He et al., 2020). This study provided a new evidence for potential therapeutic role of the GS in ischemic stroke.

Furthermore, the GS is beneficial to the restoration phase of ischemic stroke. The GS dysfunction is related to poststroke dementia (PSD), which is one of the most common and severe consequences of stroke (Pantoni, 2017). Deposition and hyperphosphorylation of tau have been implicated as the main pathophysiology underlying PSD (Zhao et al., 2014; Qiu et al., 2016). Recently, a study demonstrated that the parenchymal infiltration of CSF tracers injected into the cisterna magna was attenuated in the PSD model and that tau clearance was obstructed, suggesting that GS malfunction was a risk factor for the incidence of PSD (Back et al., 2020). Therapeutic strategies to improve the clearance of brain metabolic wastes, including tau, may be a promising approach to prevent PSD after stroke.

Collectively, the GS plays an essential role in the pathology of ischemic stroke; further studies should investigate the role of the GS in different phases of ischemic stroke so as to develop alternative treatment strategies for ischemic stroke.

## The Change in AQP4 Expression After Stroke

**FIGURE 3:**
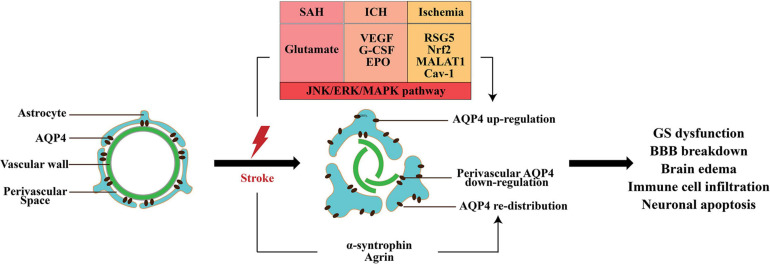
The change in AQP4 expression after stroke. AQP4 mislocalization and an increase in total AQP4 expression appear in all subtypes of stroke; however, the perivascular polarity of AQP4 decreased. The matrix constituent agrin and α-syntrophin play an important role in anchoring of AQP4 to astrocytic end-feet. After stroke, multiple factors increase the expression of AQP4, including glutamate VEGF, G-CSF, EPO, RSG5, Nrf2, lncRNA MALAT1, and Cav1. Moreover, activation of MAPK pathway is responsible for AQP4 upregulation. AQP4 mislocalization and expression change are involved in the GS dysfunction, BBB disruption, brain edema, and neuronal apoptosis induced by stroke.

Aquaporin-4, the most important molecular component in the GS, mediates the influx of CSF into the brain parenchyma and the efflux of ISF into the subarachnoid space. AQP4 is involved in multiple biological processes, including the regulation of brain edema, promotion of migration of astrocytes, calcium signal transduction, and synaptic plasticity (Chu et al., 2016). In this section, we review the expression of AQP4 in different subtypes of stroke and then examine the signaling pathways that regulate AQP4 expression and discuss therapeutic opportunities that target AQP4 (Figure 3).

### AQP4 in SAH

Previous studies have revealed that the expression level of AQP4 messenger RNA (mRNA) and protein increased at day 1 and peaked at day 3 after SAH (Long et al., 2019). One study indicated that AQP4 maintained high level even at 7 days after SAH (Luo et al., 2016).

The role of AQP4 in the pathophysiological process of SAH is complex. Several recent studies validated that AQP4 was responsible for brain edema following SAH. Zhang et al. (2020a) demonstrated that glutamate might be responsible for the elevation of AQP4 following SAH and that metabotropic glutamate receptor 1 (mGluR1) negative allosteric modulator could reduce BBB damage and cerebral edema via inhibition of AQP4. Another study found that treatment with pituitary adenylate cyclase-activating polypeptide (PACAP) inhibited the expression of AQP4 and attenuated brain edema (Fang et al., 2020). Additionally, salvinorin A and baicalin were confirmed to reduce SAH-induced brain edema via AQP4 inhibition (Sun et al., 2018; Zhang et al., 2020c). Furthermore, AQP4 inhibition preserved the function of the BBB and the GS and provided neurological benefits (Fang et al., 2020).

Some other studies reported the neuroprotective role of AQP4 in early brain injury (EBI) following SAH. Given that AQP4 facilitated ISF transport in the brain parenchyma to eliminate the toxic factors, AQP4 knockout has been shown to aggravate EBI following SAH through impairment of the GS (Liu et al., 2020a,b). Pu et al. (2019) also demonstrated that the perivascular polarity of AQP4 decreased after SAH, which resulted in accumulations of tau proteins and CD3^+^, CD4^+^, and CD8^+^ cells, and led to a series of pathological changes, including microvascular spasm, activation of glial cells, neuroinflammation, and neuronal apoptosis (El Amki et al., 2018). Meanwhile, the length density of AQP4-positive capillaries in the hippocampus was significantly reduced following SAH (Anzabi et al., 2018). One potential explanation for the controversial role of AQP4 in SAH is that the expression of AQP4 in the influx and efflux routes of the GS is different. After SAH, astrocytes surrounding the arteries were activated to increase the expression of AQP4; however, the expression of AQP4 around the veins changed slightly (Liu et al., 2020b). Therefore, the inflow of CSF from periarterial space (PAS) increased highly due to the enhanced level of AQP4, and the ISF volume expanded owing to the unchanged expression of AQP4 around the veins.

### AQP4 in ICH

It has been reported that AQP4 expression increased from 3 h after ICH and peaked between day 2 and 5 (Tang et al., 2010; Fang et al., 2015; Wang et al., 2015; Huang et al., 2020). Several studies demonstrated that VEGF, granulocyte-colony stimulating factor (G-CSF), and erythropoietin (EPO) contributed to the increase in AQP4 via the activation of the c-JUN N-terminal kinase (JNK) pathway, extracellular signal-regulated kinase (ERK) pathway, and mitogen-activated protein kinase (MAPK) pathway, respectively (Chu et al., 2013, 2014a,b).

Aquaporin-4 is involved in brain edema, BBB disruption, and neuronal apoptosis induced by ICH. It has been demonstrated that AQP4 is responsible for the explosive swelling of astrocytes and their dysfunction after ICH (Appelboom et al., 2015). Zhang et al. (2020b) observed that inhibition of the expression of AQP4 with glycyl-l-histidyl-l-lysine (GHK) could alleviate the injury of astrocytes and brain edema. Moreover, a different group also showed that AQP4 deletion reduced brain edema (Chen et al., 2020).

Several studies reported the opposite role of AQP4 in ICH. Tang et al. (2010) observed that AQP4 deletion aggravated brain edema. Meanwhile, AQP4 deletion resulted in swelling capillary endothelial cells and disruption of tight junctions (Chu et al., 2014a). Recently, in a rodent intracerebral hematoma expansion model, Chu et al. (2020) found that AQP4 deletion was associated with larger hematoma volume and more severe BBB disruption, which indicated that AQP4 reduced the hematoma volume and neurological deficits via the maintenance of BBB integrity. Furthermore, the presence of AQP4 alleviated neuronal apoptosis after ICH via inhibition of cytokines, especially TNF-α and IL-1β. These results indicate that AQP4 may have a protective effect on brain edema, BBB disruption, and neuronal apoptosis (Chu et al., 2014c). Therefore, it is difficult to draw definite conclusions about the effects of AQP4 in ICH. The mislocalization of astrocytic AQP4 may contribute to its controversial role in ICH (Qiu et al., 2015).

### AQP4 in Ischemic Stroke

Currently, a series of studies reported AQP4 expression after ischemic stroke. The continuous and dynamic observation carried out in transient MCAO demonstrated that AQP4 mRNA and protein were upregulated at 30 min after ischemia and that this lasted at least 72 h and normalized after 28 days (Badaut et al., 2007). Another study using the transient MCAO model found that there were two peaks of AQP4 expression: 1 and 48 h (Ribeiro Mde et al., 2006). AQP4 expression was positively correlated to the regulator of G protein signaling 5 (RGS5), transcriptional factor Nrf2, long non-coding RNA (lncRNA) MALAT1, and caveolin-1 (Cav-1) after ischemic stroke (Özen et al., 2018; Liu et al., 2019; Filchenko et al., 2020; Wang et al., 2020). Furthermore, MAPK pathways have been proven to play a pivotal role in AQP4 upregulation, and the activation of protein kinase C (PKC) pathway may be responsible for AQP4 downregulation (Nito et al., 2012; Wei et al., 2015).

Aquaporin-4 plays a complex bimodal function in the pathology of ischemic stroke. Most of the studies found that AQP4 inhibition, including AQP4 knockout or AQP4 gene silence using small interfering RNA (siRNA), reduced brain edema in different cerebral ischemia models. Manley et al. (2000) first showed that brain edema was decreased in AQP4 knockout mice at 24 h after permanent MCAO. Then, several studies reveal similar results (Katada et al., 2014; He and Lu, 2015; Yang et al., 2015; Yao et al., 2015; Özen et al., 2018; Liu et al., 2019; Filchenko et al., 2020; Wang et al., 2020). One study found that treatment with AQP4 inhibitor TGN-020 in MCAO mice reduced brain edema and infarct volumes through blocking fluid flow toward the parenchyma in the perivascular drainage pathways (Pirici et al., 2017). Moreover, AQP4 knockout decreased mortality, increased motor recovery, and improved long-term outcome after transient MCAO (Hirt et al., 2017).

Several studies reported the opposite role of AQP4 in the pathology of ischemic stroke. One study reported that AQP4 knockout resulted in a striking hypertrophy of astrocytes and aggravated brain injury with enlarged infarct size and a serious loss of CA1 neurons (Zeng et al., 2012). Moreover, AQP4 knockout showed more severe neutrophil infiltration and microglial activation but less astrocyte proliferation in the brain after MCAO compared to wild-type mice (Shi et al., 2012). The dual role of AQP4 in ischemic stroke is mainly due to its spatial expression heterogeneity. Studies from cerebral infarction patients demonstrated that AQP4 expression increased only in white matter, while cortical astrocytes exhibited reduced perivascular AQP4 (Stokum et al., 2015). Back et al. (2017) also found that AQP4 distribution changed from perivessel to parenchyma. Moreover, a recent study showed that impaired perivascular AQP4 covering after ischemia was associated with altered reactive astrocyte morphology and enhanced brain edema. In summary, the effects of AQP4 on the pathophysiological process of ischemic stroke are very complex, and more studies are needed to further investigate the spatial and temporal differences of AQP4 expression and its role in different phase of ischemic stroke.

## Conclusion and Perspectives

The CSF/ISF exchange and the transport systems for brain solutes and metabolic wastes have gained significant attention in recent years. Currently, the GS has become prominent in AD research by demonstrating its role in Aβ clearance. Several key proteins involved in neuroinflammation and cognitive decline, such as tau and cytokines, are also believed to be removed by the GS, supporting its potential role in the recovery stage of stroke. Information from human and rodent studies confirmed that the GS dysfunction caused a risk of stroke and was involved in the pathology of stroke. However, the molecular mechanisms of the interaction between the GS and stroke are not completely understood. First, we should clarify factors that impair or enhance the GS function in order to map the time course of pathological changes in the GS function during stroke. Second, we have already documented the existence of the GS through MRI and two-photon microscope, but novel neuroimaging modalities able to assess the function state of the GS have not yet been taken advantage of. Furthermore, high spatial resolution imaging and mathematical models are necessary to understand the CNS interstitial space. Third, it is not clear what kind of pathophysiological changes following stroke influence the redistribution of astrocytic AQP4. For instance, it is not known whether there are additional astrocytic ion channels, apart from AQP4, that facilitate the CSF/ISF exchange and metabolic wastes clearance. Finally, it has been established that glymphatic clearance is primarily active during sleep (Shokri-Kojori et al., 2018; Holth and Fritschi, 2019). Coincidentally, most stroke patients suffer from circadian rhythm disorders (Pérez-Carbonell and Bashir, 2020). Therefore, it is not clear whether improving the sleep quality of stroke patients is an effective approach to promoting recovery. In addition, some studies have shown that sleep-promoting states improved brain-wide drug distribution (Plog et al., 2018; Lilius et al., 2019). In the future, more studies should focus on the relevance of intrathecal delivery of drugs to the GS function. Anyway, a comprehensive understanding of GS will provide novel molecular markers for the prognosis and diagnosis of stroke, and promising therapeutic targets.

## Author Contributions

XZ and QH designed and conceptualized the review. TL drafted and revised the manuscript. BZ retrieved and screened the review. All authors contributed to the manuscript and approved the final version.

## Conflict of Interest

The authors declare that the research was conducted in the absence of any commercial or financial relationships that could be construed as a potential conflict of interest.
